# Secondary Degeneration of Auditory Neurons after Topical Aminoglycoside Administration in a Gerbil Model

**DOI:** 10.1155/2018/9158187

**Published:** 2018-03-01

**Authors:** Jae-Hun Lee, Min Young Lee, Phil-Sang Chung, Jae Yun Jung

**Affiliations:** ^1^College of Medicine, Dankook University, Beckman Laser Institute Korea, Cheonan, Republic of Korea; ^2^Department of Otolaryngology-Head & Neck Surgery, College of Medicine, Dankook University, Cheonan, Republic of Korea; ^3^Dankook University Hospital, Cheonan, Republic of Korea

## Abstract

Hair cells in the cochlea can be damaged by various causes. Damaged hair cells can lead to additional destruction of parts of the auditory afferent pathway sequentially, which is called secondary degeneration. Recently, researches regarding cochlear implants have been actively carried out for clinical purposes; secondary degeneration in animals is a much more practical model for identifying the prognosis of cochlear implants. However, an appropriate model for this research is not established yet. Thus, we developed a secondary degeneration model using an ototoxic drug. 35 gerbils were separated into four different groups and kanamycin was applied via various approaches. ABR was measured several times after drug administration. SGCs were also counted to identify any secondary degeneration. The results showed that outer and inner HCs were damaged in all kanamycin-treated groups. Twelve weeks after kanamycin treatment, the round window membrane injection group showed severe subject differences in hair cells and SGC damage, whereas the gelfoam group showed consistent and severe damage in hair cells and SGCs. In this study, we successfully induced secondary degeneration in hair cells in a gerbil model. This model can be used for various purposes in the hearing research area either for treatment or for preservation.

## 1. Introduction

Social impacts of hearing loss have increased in many aspects more than ever, since the prevalence of hearing loss surges in accordance with the aging process of our modern society. Noise, ototoxic drugs, infections, aging, and other diseases are responsible for cochlear end organ damage during our lifetimes. In many cases, the loss of cochlear hair cells is the main contributor to loss of sound perception. Cochlear hair cell damage can subsequently progress towards the proximal part of the auditory pathway including the nerve fiber, spiral ganglion cells (SGCs), and cochlear nucleus, which is also known as secondary degeneration [1]. This secondary degeneration shows various features in terms of the degree and rate of degeneration depending on etiologies of hair cell damage and species [2–6].

Specifically, this degeneration had been considered to be highly dependent on the status of the inner hair cell (IHC) [2, 7, 8]. Supporting cells, which are located under the inner hair cell, were also considered as an important factor that can contribute to the degree and time of secondary degeneration in both animals and humans [9, 10], and this was further supported by a study with transgenic mice [11].

Even after severe hearing loss, the degree of secondary degeneration on the remaining SGCs is very critical for hearing rehabilitation in the area of cochlear implant which is a cutting-edge modality for profound sensorineural hearing loss patients nowadays [12, 13]. Currently, cochlear implants rely on SGCs for electrical stimulation for coding of the processed acoustic sound, which means a higher hearing performance can be expected with a higher number of SGCs [14]. This urges the clinical modality to prevent or retard the secondary degeneration of SGCs while waiting for a cochlear implant surgery.

Aminoglycoside is a widely used class of antibiotics which also has ototoxicity that can induce permanent damage to the organ of Corti (OC) [15]. Particularly, kanamycin is more cochlear-toxic rather than vestibulotoxic [16] and has been used in animal research for deafening [17, 18]. Several studies tracked the histological feature of the auditory afferent pathway after kanamycin deafening [19], especially when administered with furosemide [20] that also has a potential of causing hearing loss [21]. Kanamycin can be accumulated mainly in the mitochondria of HCs [22] which can result in SGC loss by affecting the neurotrophic factors [23–25].

As described above, delaying and attenuating the timing of secondary degeneration are important for hearing rehabilitation. To investigate the therapeutic methods which can delay or prevent secondary degeneration, a stable and consistent secondary degeneration model is essentially needed. For this reason, the purpose of this study is to establish a consistent secondary degeneration model by approaching different drug treatments of kanamycin.

## 2. Methods

### 2.1. Animals

Female Mongolian gerbils (40–45 g) at 6–8 weeks of age were included in the experiment and were divided into four different groups: kanamycin via percutaneous injection (KP, *N* = 12), kanamycin at round window niche soaked in gelfoam (KG, *N* = 12), kanamycin injection through the round window membrane (RWM) (KI, *N* = 12), and control (*N* = 6). For the drug administration and hearing measurements, gerbils were anesthetized with zolazepam (Zoletil, Virbac, Carros Cedex, France) and xylazine (Rompun, Bayer, Leverkusen, Germany). All procedures were approved by the Institutional Animal Care and Use Committee of Dankook University (DKU-15-006).

### 2.2. Drug Administration

**Figure 1:**
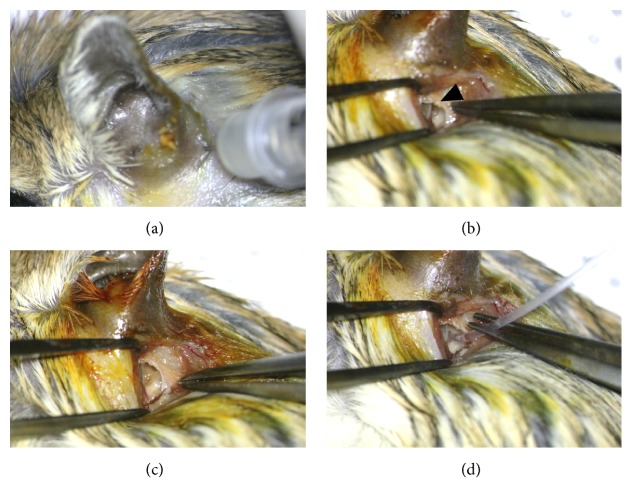
Drug delivery via different methods. The KM solution was injected by a syringe to the KP group (a). A small hole on the bulla was made and the RWM (black arrowhead) was exposed (b). A gelfoam with KM was placed on the RWM for the KG group (c). The tip of a cannula was inserted in the RWM niche for the KI group (d).

Kanamycin (KM) sulphate was diluted in normal saline (150 mg/ml) and administered in three different ways (Figure 1). For the KP group, 50 ul of the drug solution was delivered by injection with an insulin syringe (Ultrafine Insulin Syringe, Becton Dickinson, USA) to the bulla. To improve the absorption to the RWM, the animal was laid on its contralateral side, injected, and sustained for half an hour. For both KG and KI groups, the bulla was exposed through a retroauricular skin incision. After anesthetization, furs near the bulla were removed and the skin was also incised. A small hole was made on the bulla and the RWM was exposed. A small gelfoam was placed on the RWM and 4 microliters of kanamycin solution was injected with a Hamilton syringe (Hamilton Company, Nevada, USA) for the KG group. In the case of the KI group, a small hole was made on the bulla and the tip of a cannula connected with the Hamilton syringe was fitted into the RWM after the endolymph liquid was drained. Then, 4 microliters of the KM solution was gently and slowly injected. Animals in both groups were also laid on their contralateral side for surgery for stable absorption of the drug.

### 2.3. Hearing Measurement

Auditory brainstem responses (ABRs) were measured to investigate the changes of hearing threshold before and after drug administration. The evoked response signal-processing system (System III; Rucker Davis Technologies, Alachua, Florida) was adopted for ABR measurement. Animals were anesthetized with zolazepam (Zoletil, Virbac, Carros Cedex, France) and xylazine (Rompun, Bayer, Leverkusen, Germany) and were placed in a soundproof chamber. Then, needle electrodes were inserted to the vertex (as response) and ventrolateral sides of both pinnae (as reference and ground). Tone stimuli with 4, 8, 12, 16, and 32 kHz were generated from 90 dB to 10 dB in 5 dB steps and average waveforms were generated from 1024 responses. Hearing thresholds were measured before and 1, 4, and 12 weeks after drug administration.

### 2.4. Histological Analysis and Quantification

Animals were sacrificed after 4 and 12 weeks of drug administration for the histological analysis. Cochleae were harvested and fixed in 4% paraformaldehyde for 24 hours at 4°C. After fixation, the samples were decalcified with 0.5 M ethylenediaminetetraacetic acid (EDTA) for a week. Cochleae from 6 gerbils in each group were embedded in paraffin and sectioned from the apex to the basal turn to quantify the number of SGCs at 4 and 12 weeks after drug administration. Four micrometers of sectioned samples was stained with hematoxylin and eosin (H&E).

The number of SGCs was counted using ImageJ software (http://rsb.info.nih.gov/ij/). Samples were sectioned as midmodiolar plan to represent the overall place of the cochlea. SGCs in 10000 square micrometers at four different parts of the cochlea (high middle, low middle, high basal, and low basal) were counted and compared with the control group. Three or more sectioned images at each part with a 50 um interval were averaged and examined by polarizing microscopy using a BX51-P microscope (Olympus, Tokyo, Japan).

Three cochleae at each group after 12 weeks of drug administration were prepared as whole mounts and were immunostained with anti-neurofilament heavy (anti-chicken, Millipore, 1:1000) and MyosinVIIa (anti-rabbit, Millipore, 1:200). After mounting on a slide, images were taken with a confocal microscope (FV-3000, Olympus, Tokyo, Japan). 40x magnification was used and *z*-stacks were generated.

### 2.5. Statistical Analysis

Statistical analysis was performed using the Statistical Package for the Social Sciences (SPSS, Chicago, USA) software. Two-way analysis of variance (ANOVA) with Bonferroni post hoc test was adopted and significant differences were determined when the *p* value was <.05.

## 3. Results

### 3.1. Serial Changes of ABR Thresholds

**Figure 2:**
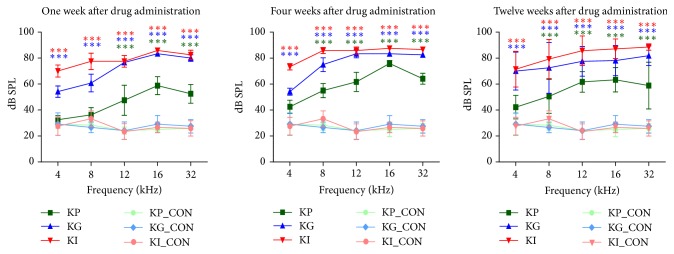
Serial changes of the hearing threshold after drug administration. Hearing thresholds at multiple frequencies were measured at three different time points (1 week, 4 weeks, and 12 weeks) for kanamycin percutaneous (KP), kanamycin gelfoam (KG), and kanamycin RW injection (KI) groups. See Methods for more descriptions. In the KP group, hearing thresholds increased at high frequency regions (12, 16, and 32 kHz) at four weeks after drug administration and these increased thresholds remained until 12 weeks after drug administration. KG group showed a more severe hearing threshold change at all time points. KI group showed the worst hearing thresholds change at all tested frequencies and all test points (^*∗∗∗*^*p* < 0.001).

We tested different delivery techniques (single application) to induce hearing loss and evaluated the hearing outcome. ABR was measured at three time points in all groups, one, four, and twelve weeks after the drug administration. Hearing threshold shifts were observed in all groups at any given time points (Figure 2). At the one-week time point, hearing thresholds of all kanamycin injected groups were statistically different from the control (two-way ANOVA, df = 3, *F* = 193.1, *p* < .0001). In the KP group, post hoc *t*-test revealed that statistical significance exists at 12, 16, and 32 kHz (12 kHz: *p* < .001, 16 kHz: *p* < .001, 32 kHz: *p* < .001). As with the KG group, hearing thresholds were significantly elevated at all test frequencies (*p* values of post hoc test: 4 kHz < .001, 8 kHz < .001, 12 kHz < .001, 16 kHz < .001, and 32 kHz < .001). Similarly, the KI group showed a significant difference at all test frequencies (*p* values of post hoc test: 4 kHz < .001, 8 kHz < .001, 12 kHz < .001, 16 kHz < .001, and 32 kHz < .001) (Figure 2).

According to the results of this part, using the local delivery techniques to induce a substantial hearing threshold shift with a single application was possible. Threshold changes of the KG and KP groups showed increased hearing threshold shifts at higher frequencies due to the closer location to the round window where the drug is presumably delivered. In the KI group, most of the deterioration in the hearing threshold was observed; however, the invasiveness of the delivery technique cannot be disregarded.

At the 4-week time point, hearing thresholds of all kanamycin injected groups were statistically different from the control (two-way ANOVA, *F* < 326.9, df < 3, *p* < .0001). Post hoc *t*-test revealed that all three groups showed statistically significant threshold shifts at all the tested frequencies (KP group: 4 kHz < .001, 8 kHz < .001, 12 kHz < .001, 16 kHz < .001, and 32 kHz < .001; KG group: 4 kHz < .001, 8 kHz < .001, 12 kHz < .001, 16 kHz < .001, and 32 kHz < .001; KI group: 4 kHz < .001, 8 kHz < .001, 12 kHz < .001, 16 kHz < .001, and 32 kHz < .001). These threshold shifts were constant until 12 weeks after the drug administration while the KP group showed a little recovery at 4 kHz (*p* < .01).

With a single application using a different delivery technique, hearing threshold change was maintained until the 12-week time point. This result suggests that the hearing deterioration observed at the 1-week time point is not transient but is permanent, possibly due to the irreversible loss and not the temporary damage of the hair cells.

### 3.2. Hair Cells and Neurofilaments Damage Was Variable Depending on the Drug Administration Methods

**Figure 3:**
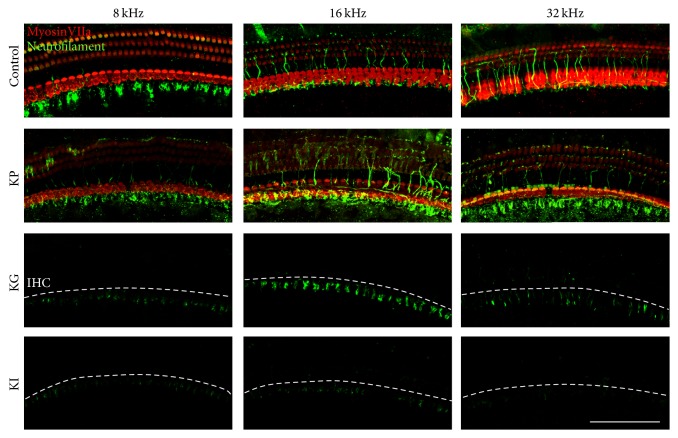
Epifluorescence analysis of the OC at 12 weeks after drug administration. Hair cells (MyosinVIIa, red) and the peripheral auditory nerve (Neurofilament, green) were observed at three parts of the cochlea that are tonotopically responsible for 8, 16, and 32 kHz of hearing. The control group showed intact hair cells and nerve fibers. KP group showed intact IHCs and a few defects of OHCs at three different parts. Nerve fiber connections from hair cells to SGCs were disrupted in the KP group. In the KG and KI groups, IHCs and OHCs completely disappeared and only fragments of nerve fibers were observed. The three groups were kanamycin percutaneous (KP), kanamycin gelfoam (KG), and kanamycin RW injection (KI). The white dotted lines represent the IHC place. Scale bar is 100 um.

A decrement in hearing threshold after drug administration would be highly related to the status of the OC, especially hair cells (HCs) and nerve fibers connected to them. Thus, we investigated the status of the HC and nerve fibers by immunostaining with whole mount preparation. The status of HCs and nerve fibers at the apex, middle, and base parts of the cochlea, which represents 8, 16, and 32 kHz, was identified. After twelve weeks of drug administration, the KG and KI groups showed a total loss of HCs and a partial loss of nerve fibers at three selected parts of the cochlea. In the case of the KP group, we found that the status of the nerve fiber and HCs was preserved (Figure 3). These results suggest that the HC and nerve fiber can be severely damaged by a single application of kanamycin depending on the delivery method, and such damage would cause a permanent threshold shift in the KG and KI groups. In the case of the KP group, a threshold shift was maintained for 12 weeks without anatomical change in the HCs and nerve fibers.

### 3.3. OC Was Damaged over Time after Drug Administration

**Figure 4:**
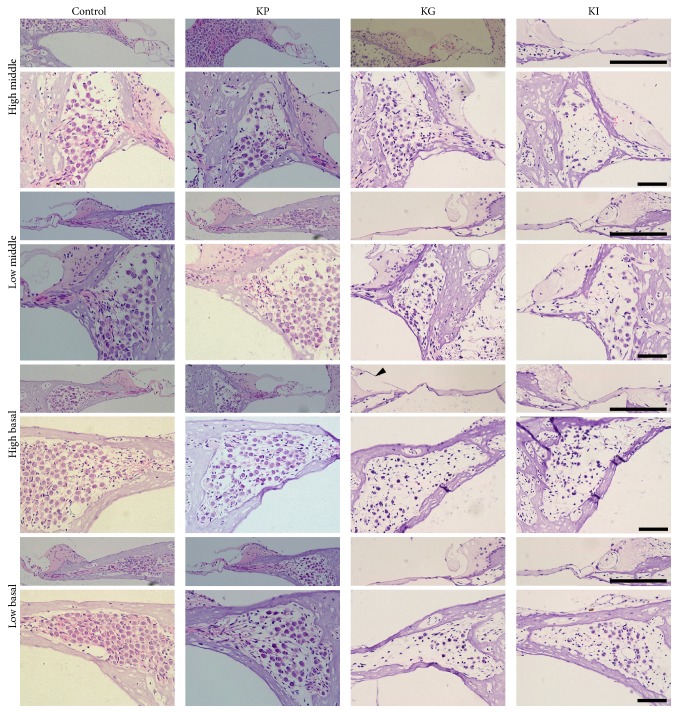
Histologic analysis of the OC and SGCs at four different parts of the cochlea at 4 weeks after drug administration. At 4 weeks after drug administration, OC was intact in both KP and KG groups, but the KG group showed a flat epithelium without any sensory cells at all parts except the high middle part. In the KG group, a damaged spiral limbus (black arrowhead) was found at the high basal part. The KI group also showed severely damaged OC at all parts of the cochlea. The KG group showed no reduction of SGC density, showing a similar result to the control group. The KG group showed sparse SGCs at low middle, high basal, and low basal parts of the cochlea. The KI group showed sparse SGCs at all parts of the cochlea. The three groups were kanamycin percutaneous (KP), kanamycin gelfoam (KG), and kanamycin RW injection (KI). Scale bar represents 100 um.

**Figure 5:**
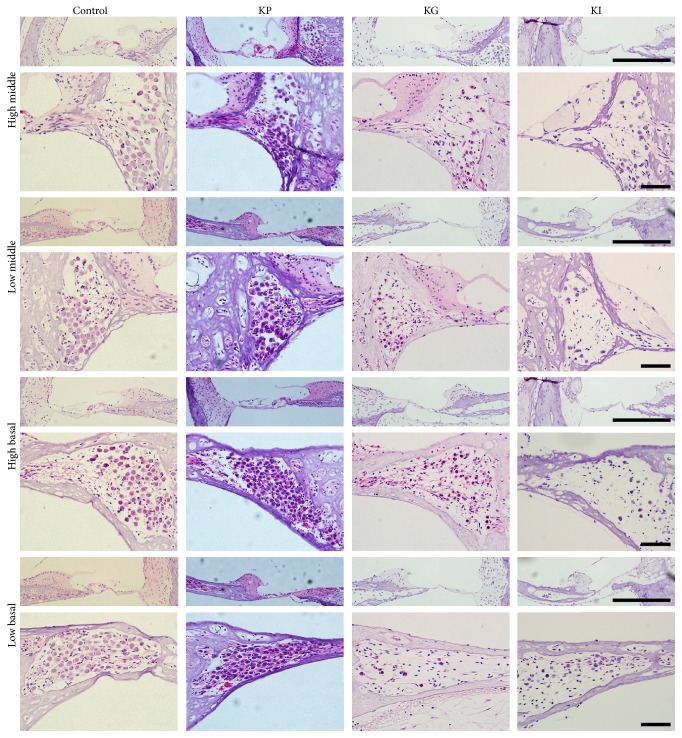
Histologic analysis of the OC and SGCs at four different parts of the cochlea at 12 weeks after drug administration. At 12 weeks after drug administration, the OC was intact in the KP group, but the KG group showed a flat epithelium without any sensory cells at all parts except the high middle part. The KI group showed a disrupted OC at all parts of the cochlea. The KP group showed no reduction of SGC density, showing a similar result to the control group. However, the KG group showed sparse SGCs at all parts of the cochlea, which is more severe than 4 weeks after drug administration. For the KI group, more severe depletion of SGCs was found at all parts of the cochlea. The three groups were kanamycin percutaneous (KP), kanamycin gelfoam (KG), and kanamycin RW injection (KI). Scale bar represents 100 um.

According to the immunostaining results, kanamycin causes damage not only to the HCs in the OC, but also to the auditory nerve fibers. To investigate the degree of degeneration in the OC and the possibility of additional degeneration of the auditory ascending pathway, the status of the OC within the sectioned images was identified. Four locations in the cochlea were selected as representative areas (Figures 4 and 5). After four weeks of drug administration, the OC was intact at the four selected locations in the control and KP groups. In the KG group, the OC was damaged and showed a flat epithelium at both high and low basal parts of the cochlea. In the case of the KI group, the OC was also damaged and showed a flat epithelium throughout the cochlea (Figure 4). This result confirmed that a single administration of kanamycin can cause damage to HCs at 4 weeks after the treatment depending on the administration method.

After twelve weeks of drug administration, still, the status of the OC was intact at all locations in the control and KP groups. In the KG group, damage to the OC was extended to the upper parts of the cochlea, but the high middle part of the OC was undamaged. In the KI group, the status of the OC was varied depending on the subject. One subject showed a total loss of the OC at all the selected parts, whereas two subjects showed an intact status of the OC. These results suggest that the kanamycin solution injected through the RWM would have leaked out if the perilymph was not well flushed.

### 3.4. SGCs Were Damaged following Hair Cell Loss

**Figure 6:**
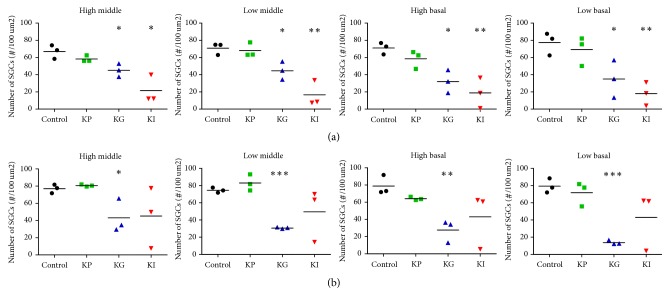
SGC densities at four different parts of the cochlea at 4 (a) and 12 weeks (b) after drug administration. The KP group showed no significant decrease in SGC densities compared to the control at both 4 and 12 weeks. The KG group showed a significant SGC density decrease at all locations at both time points. The KI group showed a significant SGC density decrease at all locations at the 4-week time point but did not show a significant decrease at the 12-week time point. All units in the plot (black dot: control; green square: KP; blue triangle: KG; red inverted triangle: KI) represent individual subjects. The three groups were kanamycin percutaneous (KP), kanamycin gelfoam (KG), and kanamycin RW injection (KI). The asterisk (*∗*) indicates a *p* value lower than .05, the double asterisk (*∗∗*) indicates a *p* value lower than .01, and the triple asterisk (*∗∗∗*) indicates a *p* value lower than .001.

The status of SGC was also observed for identifying additional damage in the auditory pathway. Similar to the OC, SGCs at four locations were counted and compared with the control group (Figures 3 and 4). In the KP group, there were no difference in the density of the SGC at four and twelve weeks after drug administration (Figure 6). However, the number of SGCs in the KG group was significantly decreased compared with the control group at four weeks after drug administration (Figure 6(a)), and these decrements were increased at twelve weeks after drug administration (Figure 6(b)). In the KI group, the number of SGCs was significantly decreased in all selected locations at four weeks after drug administration (Figure 6(a)). However, after twelve weeks of drug administration, the number of SGCs was not consistent between subjects and a huge subject difference existed and was not significantly different from the control group (Figure 6(b)). These results suggest that a single treatment of kanamycin can damage HCs and this HC loss causes deterioration at the upper part of the auditory pathway.

## 4. Discussions

### 4.1. KM Ototoxicity

Kanamycin is a well-known ototoxic agent, and it is a widely used model to mimic human sensorineural hearing loss with various delivery methods in an animal model [20, 26–29]. However, to induce substantial hearing loss in a rodent model, multiple injections or other drug combinations are required. Several previous researches used kanamycin as an ototoxic drug combined with other agents for achieving total loss of HC which is incomplete with kanamycin alone [5, 30]. In particular, furosemide, which can manipulate the blood labyrinth barrier, has been used as a combination agent and showed better ototoxic damage compared to kanamycin alone. However, this agent is a diuretic and can systemically affect the whole body. This untargeted effect would cause changes inside the cochlea, resulting in ambiguous toxic or inflammatory damage. It was reported that furosemide may itself cause a hearing loss by inducing transient malfunction in the stria vascularis [31, 32]. Therefore, we used a very high concentration of kanamycin and applied it directly to the round window membrane with a gelfoam and acquired severe SGN loss at 4 weeks after drug administration. This high concentration of kanamycin was two or three times higher than in previous studies [5, 30] and causes total loss of both hair cells and SGCs at 12 weeks after a single treatment.

The ototoxicity of kanamycin is a well-known issue throughout the clinical and animal research. It is reported that kanamycin induces production of reactive oxygen species and these attack the cochlear hair cells, which is an irreversible injury, resulting in a hearing disorder (Jiang et al., 2005). We did not explore the mechanism of kanamycin toxicity at the HC and SGC in this study. Nevertheless, we might expect that the same ototoxic mechanism would be involved in this study.

### 4.2. Gerbil as a Proper Hearing Research Model

The Mongolian gerbil is a well-established animal model for hearing research [33]. Since the range of audible frequency is more similar to that of humans than other rodents such as mice, rats, or guinea pigs [34], gerbils have been considered as a suitable model in hearing research including aminoglycoside toxicity [5]. Furthermore, the larger bulla and thin skull enable a surgical approach to the cochlea. In our study, we applied various approaches to treat with aminoglycosides and acquired consistent results after surgery with the aforementioned reasons except with the RWM injection group. Additionally, due to the location of the stapedial artery that is not blocking the round window, gerbils have been widely used as an animal model for CI studies. Moreover, the high reproduction rate and easier breeding and handling make gerbils more appropriate for animal studies. Their characteristics (i.e., they were born deaf and have a late onset of hearing [35]) boost the versatility for various approaches that are possible with this animal model. All in all, we suggest that gerbils can serve as a proper animal model in various hearing research areas.

### 4.3. Drug Delivery Agent

We applied various ways of drug administration to find the most appropriate way that can induce secondary degeneration. According to our results, the RWM injection showed a more dramatic change in the OC within a short time than the gelfoam group. The number of SGCs was severely decreased at four selected parts of the cochlea at 4 weeks after kanamycin treatment through the RWM injection. However, a prominent subject difference also existed with this approach. During treatment, the KM solution could not disperse well because of the pressure inside the scala tympani, and it also leaked out right after injection using a cannula. Together with these, we considered that KM RWM injection is not an appropriate way to create a secondary degeneration model (Figure 6). Otherwise, KM application with gelfoam showed very consistent and effective results within subjects compared to any of the other methods (Figure 6).

Drug delivery agents have been studied in the otology research area for ototoxic or therapeutic purposes. It has been reported that gelfoam increases the effect of a drug itself by allowing the drug to be retained longer at the target area. Abbas and Rivolta (2015) used aminoglycosides with a gelatin sponge and reported a more significant change in the hearing threshold after 2 weeks of treatment than with KM alone [5]. However, when they applied gentamycin to the RWM with a gelatin sponge, which is also a well-known ototoxic drug, it did not cause hearing loss. They explained that this is due to the polar nature of the gelatin sponge which blocked the penetration of gentamycin into the RWM [5]. Poloxamer 407 has also been used as a delivery agent, including nanoparticles and an ototoxic drug, which can provide sustained release at the target area [36, 37]. Together with these, we thought that kanamycin treatment with poloxamer 407 would also be a proper way to create a secondary degeneration model in gerbils.

### 4.4. Secondary Degeneration Modeling and Possible Treatment

For generating a secondary degeneration model for diverse purposes, various methods would be applicable in animal research. A recent study that investigated KM toxicity reported that KM did not damage adult spiral ganglion neurons [4]. In this study, hair cells and ganglion neurons in postnatal day 3 rat cochlear organ culture were damaged by KM, whereas there was no toxicity in adult rat ganglion cells in an organotypic culture model. This result supports the notion that the degeneration of SGCs in our study was damaged not by KM itself but influenced from the loss of HC as secondary damage. It also supports the fact that our reproducible secondary degeneration model with the gelfoam approach is suitable for a secondary degeneration model.

As seen in Figure 3, the KP group showed an intact histological structure of HCs and neurofilaments, even with a hearing threshold decrement 12 weeks after drug administration. This result suggests the possibility that secondary degeneration could be initiated at the upper level of the HC. It was reported that a low dose of aminoglycoside causes synaptic changes without HC loss [38]. If the secondary degeneration starts from the damage synapse level which is the most vulnerable factor in the cochlea, neurotrophin and photobiomodulation would be appropriate therapeutic approaches. Neurotrophin factor, especially NT-3, was reported as a very useful agent which can protect against synaptic loss due to noise exposure [36, 39]. Photobiomodulation with a low-level laser (LLL) has also been studied in hearing research areas, and it was reported that it has a protective effect against HC after noise exposure [40, 41]. Furthermore, it has a neuroprotective effect against Ouabain on SGC [42]. We will try to protect or delay the secondary degeneration with either neurotrophin or photobiomodulation in the near future.

## 5. Conclusion

We induced secondary degeneration of HCs in a gerbil model through diverse drug delivery approaches in this study. High concentrations of kanamycin application with gelfoam on the RWM caused severe HC loss and this extended to degeneration of the auditory nerve and SGCs. This model can be used for various purposes in the hearing research area either for treatment or for preservation. Furthermore, this model would be applicable for research regarding cochlear implants.
